# The Association of a Positive Fecal Immunochemical Test With the Risk of Gastroesophageal Cancer: An Age‐Sex‐*H. Pylori* Exposure Matched Cohort Study and Cost‐Effectiveness Analysis

**DOI:** 10.1111/hel.70120

**Published:** 2026-03-23

**Authors:** Zohar Levi, Naim Abu‐Freha, Doron Boltin, Maya Aharoni Golan, Tom Konikoff, Orly Sneh Arbib, Rachel Gingold Belfer, Sapir Eizenstein, Alex Vilkin, Shiri Kusnir, Adi Turgeman, Tanya Babich, Moshe Leshno, Anath A. Flugelman, Hadar Edelman‐Klapper, Elizabeth Half‐Onn

**Affiliations:** ^1^ Division of Gastroenterology Rabin Medical Center Petach‐Tikva Israel; ^2^ Faculty of Medical and Health Sciences Tel‐Aviv University Tel Aviv Israel; ^3^ Department of Gastroenterology Soroka Medical Center Beer Sheba Israel; ^4^ Ben‐Gurion University Beer Sheba Israel; ^5^ Kaplan Medical Center Rehovot Israel; ^6^ Faculty of Medicine The Hebrew University of Jerusalem Jerusalem Israel; ^7^ Davidoff Cancer Center Petach‐Tikva Israel; ^8^ Research Authority Rabin Medical Center Petach Tikva Israel; ^9^ Cooler School of Management Tel‐Aviv Israel; ^10^ Technion‐Israel Institute of Technology Haifa Israel; ^11^ Department of Gastroenterology Rambam Medical Center Haifa Israel

**Keywords:** colorectal cancer (CRC), cost‐effectiveness, fecal immunochemical test, gastroesophageal cancer, screening

## Abstract

**Background and Aims:**

We evaluated the association between Fecal Immunochemical Test (FIT) results and the risk of gastroesophageal cancer (GEC) in a matched cohort, as well as the cost‐effectiveness of a one‐time esophagogastroduodenoscopy (EGD) for individuals who tested FIT‐positive.

**Methods:**

We formed a cohort of individuals aged 50–75 years who underwent FIT testing at Clalit Health in Israel from 2016 to 2019. For each person with a positive FIT result, we matched three individuals with negative results by age, gender, and *H. pylori* exposure. We used adjusted hazard ratios (adjHRs) to assess the association between a positive FIT result and the risk of GEC within 36 months. We calculated the incremental cost‐effectiveness ratio (ICER) for a one‐time EGD costing USD 350 in individuals who tested positive for FIT, and considered it cost‐effective if below USD 50,000.

**Results:**

The study included 150,391 individuals (47.6% female, median age 62.4 years). During follow‐up, 202 cases of GEC were recorded: 0.17% in FIT‐positive individuals (64/37,709) and 0.12% in FIT‐negative individuals (138/112,682), adjHR 1.39 (95% CI 1.03–1.87). GEC was also associated with *H. pylori* exposure (adjHR 1.43, 95% CI 1.08–1.90) and immigration from high‐risk countries. A one‐time EGD demonstrated favorable cost‐effectiveness across various scenarios, with an ICER of USD 25,535/QALY.

**Conclusions:**

This matched‐cohort study suggests that individuals with a positive FIT may have an increased risk of GEC, comparable to that of established high‐risk populations. Adding a one‐time EGD to colonoscopy for FIT‐positive individuals may be a cost‐effective approach for healthcare systems that can accommodate such interventions.

## 1 Introduction

The European Society of Gastrointestinal Endoscopy (ESGE) recommends gastric cancer screening for high‐risk populations, and in areas with intermediate risk, screening may be appropriate depending on local conditions and endoscopic capacity [1]. Similarly, the American Gastroenterological Association (AGA) suggests that screening may be considered for immigrants from high‐incidence regions and other high‐risk groups [2]. A recent systematic review and meta‐analysis further concluded that esophagogastroduodenoscopy (EGD) can play a valuable role in early detection of gastric cancer in intermediate‐risk countries, particularly when integrated with colorectal cancer (CRC) screening efforts [3]. As fecal immunochemical test (FIT) testing is regarded as insensitive to bleeding from the upper gastrointestinal tract, current guidelines do not recommend an upper endoscopy for patients with a positive FIT and a negative colonoscopy [4, 5, 6, 7, 8, 9, 10]. A recent large‐scale study from the Netherlands reported that FIT‐positive individuals had an increased risk of cancers proximal to the colon (HR 1.55; 95% CI, 1.44–1.67). However, as the 3‐year cumulative incidence remained below 1%, the authors concluded that EGD is not justified for all FIT‐positive individuals in Western populations [11]. A study from the United States supported these findings [12]. A key limitation of these studies is potential bias arising from differences between FIT‐positive and FIT‐negative groups, as well as confounding factors such as *Helicobacter pylori* exposure and limited generalizability to diverse populations. Israel represents a unique setting for further evaluation, as approximately 15% of the population are immigrants from the former Soviet Union, a region considered high risk for gastric cancer [13, 14]. In this context, the current study assessed the association between FIT results and the risk of gastroesophageal cancer (GEC) in a matched cohort, controlling for age, sex, and *H. pylori* exposure. We also evaluated the cost‐effectiveness of a one‐time EGD for FIT‐positive individuals compared with no screening.

## 2 Methods

### 2.1 Study Population and Design

We established a matched cohort of individuals aged 50–75 years who underwent FIT at Clalit Health Services, Israel, between 2016 and 2019. For each FIT‐positive individual, three FIT‐negative individuals were matched based on age, sex, and *Helicobacter pylori* exposure. Exposure was defined as either a positive laboratory test (stool antigen or urea breath test) or receipt of an eradication regimen specific for *H. pylori* . To minimize confounding between FIT‐positive and FIT‐negative individuals differing in age and sex, we applied age‐ and sex‐matched pairs. To reduce confounding in gastric cancer, matching was additionally performed based on *H. pylori* status. As *H. pylori* is not an established causal determinant of esophageal or small bowel cancers, this matching step does not adversely affect comparability for these malignancies. Such outcome‐specific confounder matching is consistent with contemporary recommendations for observational study design, whereby covariates are matched when they influence the exposure–outcome relationship for at least one component outcome without introducing bias in others [15]. The source cohort included 278,729 individuals who underwent FIT testing during the study period. FIT‐positive individuals were matched to FIT‐negative individuals at a 1:3 ratio on age, sex, and *H. pylori* status. A 1:3 ratio was selected to improve statistical efficiency, as precision gains beyond 1:3–1:4 matching are minimal [16], and larger ratios were not feasible without compromising matching quality or excluding FIT‐positive cases. Exclusion criteria included lack of continuous membership in Clalit for ≥ 5 years before the index FIT date and a prior diagnosis of gastrointestinal cancer (ICD codes C15–C21). Subjects were monitored for CRC (C18–C20), gastroesophageal cancer (GEC; C15–C16), and small bowel cancer (C17) for up to 36 months after the index FIT or until death. Incident colorectal cancers were excluded prior to estimating hazard ratios (HRs) for GEC. The study protocol was approved by the Clalit Health Institutional Review Board and exempted from informed consent (RMC 0122–20).

### 2.2 Data Source

Data were obtained from the Clalit Health database, the largest among Israel's four integrated healthcare organizations, covering 4.7 million members (53% of the population). The database includes detailed demographic information, laboratory results, clinical diagnoses, and medication dispensing data [17]. Electronic prescription and pharmacy data, validated since 2005, are highly accurate as medications are dispensed at minimal cost and linked to medical records [18]. Cancer diagnoses were retrieved from the Israel Cancer Registry, which maintains > 95% accuracy and international coding standards [19].

### 2.3 Variables

Collected variables included: FIT result (negative vs. positive, threshold ≥ 15 μgHb/g; Sentinel, Milan, Italy), age, sex, country of birth (Israel, former USSR, Asia, Africa, Europe), and socioeconomic status (SES; high, medium, low), defined by the Israel Central Bureau of Statistics [20]. Other variables included smoking status (current/past vs. never), anemia within 6 months before FIT (Hb < 13.2 g/dL in men, < 11.6 g/dL in women), *H. pylori* exposure (laboratory‐confirmed or eradication therapy with clarithromycin–amoxicillin–PPI, clarithromycin–levofloxacin–PPI, or tetracycline/doxycycline–tinidazole/metronidazole–PPI [18]). Non‐tested individuals were grouped with *H. pylori* negatives, as only 8% of nonexposed individuals were formally tested. Medication exposures within 6 months before FIT included aspirin, NSAIDs, antiplatelets, anticoagulants, and PPIs, classified by the Anatomical Therapeutic Chemical (ATC) system.

### 2.4 Cost‐Effectiveness Analysis

We developed a Markov microsimulation model to evaluate the cost‐effectiveness of one‐time EGD following a positive FIT compared with no additional screening. The target population consisted of FIT‐positive individuals aged 50–75 years, matching our cohort characteristics (median age 65 years, 48.5% male). Model transitions included localized, regional, and metastatic disease [21, 22, 23]. Outputs included GEC deaths, quality‐adjusted life years (QALYs), costs, and resource utilization for hypothetical cohorts of 10,000 individuals [24]. Costs and QALYs were discounted at 3% annually [24]. Cost‐effectiveness was expressed as the incremental cost‐effectiveness ratio (ICER), benchmarked against Israel's GDP per capita (139,600 NIS; USD 43,600 in 2020 [25]) and WHO thresholds [26, 27]. Screening was considered cost‐effective at ICER<USD 50,000 [22, 23]. Given established evidence supporting surveillance cost‐effectiveness once gastric intestinal metaplasia is diagnosed [3], our analysis focused exclusively on initial screening. Treatment costs reflected Israeli Ministry of Health pricing: localized cancer USD 23,570, regional USD 51,410, metastatic USD 69,160 [28]. EGD cost was USD 350 (Israeli Ministry of Health [28]). Quality‐adjusted life years (QALYs) were calculated using accepted rates [healthy (1.00), dysplasia (0.98), localized cancer (0.88), regional cancer (0.77), metastatic cancer (0.68)] [3]. We applied 3% annual discount and a lifetime horizon (100 years). The model assumed 50% of screen‐detected cancers were localized, but included a sensitivity analysis for alternative stage distributions.

### 2.5 Sensitivity Analyses

Generalizability: EGD costs varied from USD 200–1000, and treatment costs ±50% to assess transferability across healthcare systems. Scenario Analyses: Examined (1) high‐risk population ( *H. pylori* ‐positive immigrants), (2) age cohorts by 5‐year intervals, (3) gastric cancer only, (4) alternative stage distributions. One‐Way Analyses: Each parameter was varied individually across plausible ranges: localized disease, treatment costs (±50%), discount rate (0%–5%), time horizon (80–100 years), age (50–70 years).

### 2.6 Statistical Analysis

The association between FIT status and GEC risk was evaluated using time‐to‐event models. Kaplan–Meier curves and log‐rank tests were used to estimate the time from FIT to diagnosis. For non‐cancer cases, follow‐up was censored at death or study end. Cox proportional hazards models, adjusted for covariates and tested for proportionality using log‐minus‐log plots, were applied. Results are presented as adjusted HRs with 95% confidence intervals (CIs), accounting for death as a competing risk [29]. Sensitivity analyses were performed in subgroups without anemia and by *H. pylori* status. SES data were missing in 6% of participants and treated as a separate category. Analyses were performed with SAS version 8.3 (SAS Institute, Cary, NC, USA).

## 3 Results

### 3.1 Cohort Characteristics

**FIGURE 1:**
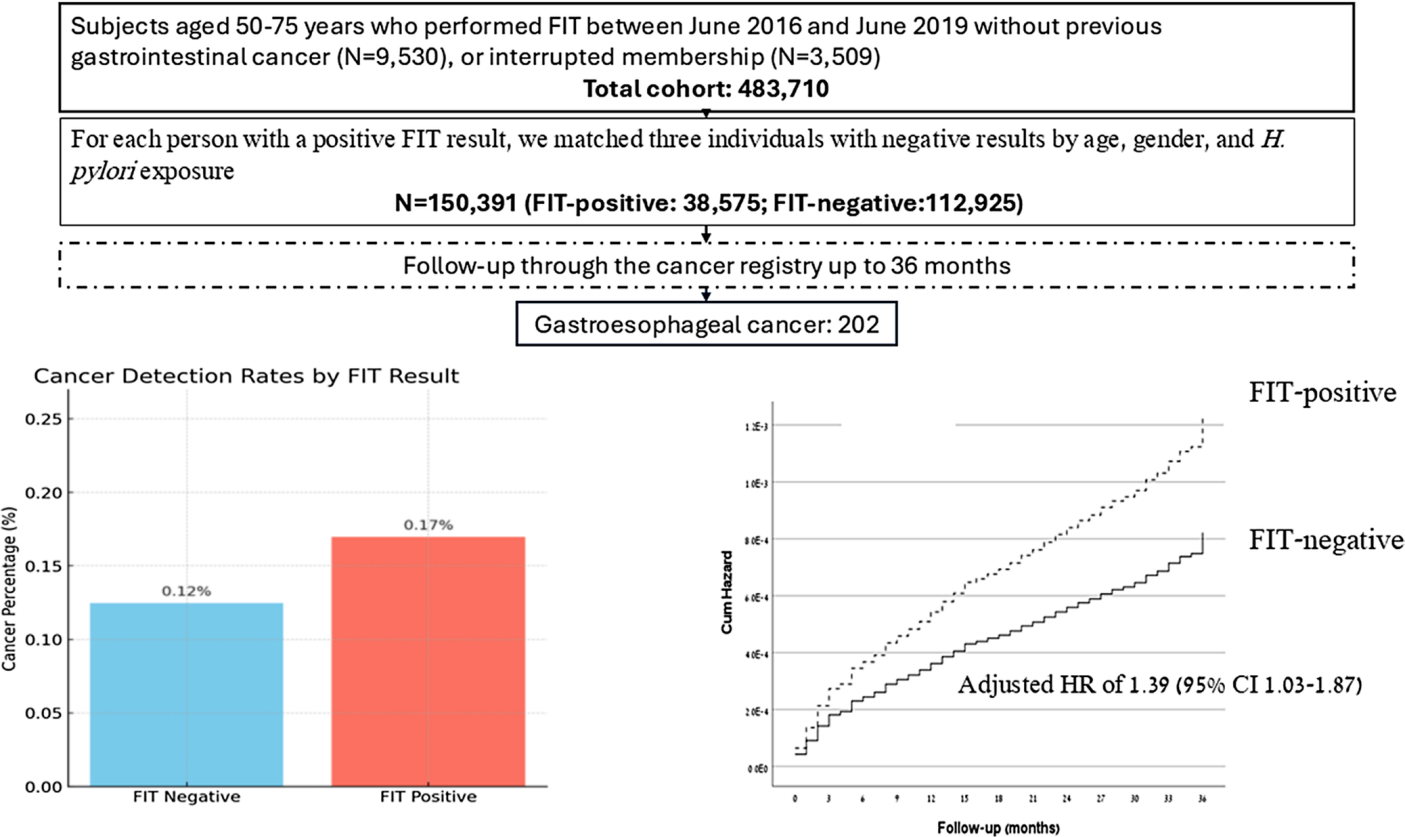
Flowchart of the study.

From the initial population, 9530 individuals were excluded due to a prior diagnosis of gastrointestinal cancer, and 3509 due to a lack of continuous enrollment in Clalit Health. The primary analytic cohort included 483,710 individuals, of whom 38,599 (8.0%) had a positive FIT. After excluding 1109 incident CRC cases, the final matched cohort consisted of 150,391 individuals (47.6% female; median age, 62.4 years) (Figure 1). Of these, 37,709 were FIT‐positive, and 112,682 were FIT‐negative (Table 1). The majority of participants (61.0%) were born in Israel, while 13.4% were born in the former USSR. Overall, 43.7% were current or past smokers, 30.1% had documented *H. pylori* exposure, and 16.0% presented with anemia within 6 months of FIT testing. Regarding medications, 22.1% were current PPI users, 28.2% were using aspirin, 20.1% were using NSAIDs, and 6.5% were on antiplatelet or anticoagulant therapy. As the cohort was matched for age, sex, and *H. pylori* exposure, no differences in these variables were observed between FIT‐positive and FIT‐negative groups.

**TABLE 1:** Cohort characteristics (each FIT‐positive individual was matched with three FIT‐negative individuals by age, sex, and *H. pylori* exposure).

	Total, *N* (%)	FIT positive; *N* (%)	FIT negative; *N* (%)	Δ
**Total**	150,391 (100.0%)	37,709 (100.0%)	112,682 (100.0%)	
**Age, years**				
Median, IQR	62.5 (56.3–67.9)	62.5 (56.4–67.9)	62.5 (56.3–68.0)	Matched variable
**Sex**				
Women	71,507 (47.5%)	17,785 (47.2%)	53,722 (47.7%)	Matched variable
Men	78,884 (52.5%)	19,924 (52.8%)	58,960 (52.3%)	Matched variable
***H. pylori***				
Non‐tested/negative	105,100 (69.9%)	26,307 (69.8%)	78,793 (69.9%)	Matched variable
Exposed	45,291 (30.1%)	11,402 (30.2%)	33,889 (30.1%)	Matched variable
**Country of birth**				
Israel	91,777 (61.0%)	22,801 (60.5%)	68,976 (61.2%)	−0.7%
Immigrant Former USSR	20,180 (13.4%)	5489 (14.6%)	14,691 (13.0%)	1.6%
Immigrant Africa	18,503 (12.3%)	4597 (12.2%)	13,906 (12.3%)	−0.1%
Immigrant Asia	10,258 (6.8%)	2431 (6.4%)	7827 (6.9%)	−0.5%
Immigrant Europe	9673 (6.4%)	2391 (6.3%)	7282 (6.5%)	−0.2%
**Socioeconomic state**				
High	23,853 (15.9%)	5110 (13.6%)	18,743 (16.6%)	−3.0%
Medium	96,445 (64.1%)	24,600 (65.3%)	71,845 (63.8%)	1.5%
Low	30,302 (20.0%)	8168 (21.2%)	22,134 (19.6%)	1.6%
**Smoking category**				
Never	84,722 (56.3%)	19,708 (52.3%)	65,014 (57.7%)	−5.4%
Current/past	65,669 (43.7%)	18,001 (47.7%)	47,668 (42.3%)	5.4%
**Anemia** ^a^	24,142 (16.0%)	7039 (18.7%)	17,103 (15.2%)	3.5%
**Proton pump inhibitors** ^a^	33,223 (22.1%)	9331 (24.7%)	23,892 (21.2%)	3.5%
**Aspirin** ^a^	42,342 (28.2%)	12,046 (31.9%)	30,296 (26.9%)	5.0%
**NSAIDs** ^a^	30,226 (20.1%)	8165 (21.6%)	22,061 (19.6%)	2.0%
**Anti‐platelet/coagulants** ^a^	9766 (6.5%)	3312 (8.8%)	6454 (5.7%)	3.1%

Abbreviations: FIT, fecal immunochemical test; IQR, interquartile range; NSAIDS, non‐steroidal anti‐inflammatory drugs; USSR, Union of Soviet Socialist Republics.

^a^ Within 6 months before the FIT date. *H. pylori* exposure was defined as either a positive stool antigen, a positive breath test, or receipt of a specific anti‐ *H. pylori* treatment.

### 3.2 Factors Associated With Gastroesophageal Cancer Risk

In this matched cohort of 150,391 participants, 202 individuals were diagnosed with gastroesophageal cancer (173 gastric cancer cases; 29 esophageal cancer cases) during follow‐up (Table 2). Patients who developed GEC were older at baseline (median age 67.0 years) compared with those who remained cancer‐free (62.3 years). GEC was diagnosed in 64 of 37,709 FIT‐positive individuals (0.17%) and in 138 out of 112,682 FIT‐negative individuals (0.12%), corresponding to an adjusted hazard ratio (HR) of 1.39 (95% CI, 1.03–1.87) (Figure 1). *H. pylori* exposure was also associated with an increased risk (HR 1.43; 95% CI, 1.08–1.90). Immigration status was a significant determinant of risk. Individuals from the former Soviet Union demonstrated the highest risk (adjusted HR 2.46; 95% CI, 1.73–3.49), followed by immigrants from Asia (HR 2.20; 95% CI, 1.38–3.52) and Europe (HR 2.13; 95% CI, 1.31–3.46). Male sex was associated with a 2.48‐fold higher risk than female sex (95% CI, 1.82–3.40). Current or past smoking was associated with an 85% increased risk relative to never smoking (HR 1.85; 95% CI, 1.40–2.45). Among clinical factors, anemia within 6 months of FIT testing was associated with a twofold higher risk (HR 2.01; 95% CI, 1.48–2.74). Proton pump inhibitor use was also associated with an increased risk (HR 1.52; 95% CI, 1.13–2.07).

**TABLE 2:** Factors associated with the risk of gastroesophageal cancer in a matched cohort by age, sex, and *H. pylori* exposure.

Factor	No cancer	Cancer	Dead	Adjusted HR (95% CI), *p*
**Total**	146,914 (100.00%)	202 (100.00%)	3275 (100.00%)	
**FIT result**				
Negative	110,511 (92.20%)	138 (0.12%)	2033 (1.80%)	1.00 (1.00–1.00)
Positive	36,403 (99.80%)	64 (0.17%)	1242 (3.20%)	1.39 (1.03–1.87), 0.030
**Age, median (IQR)**	62.3 (56.3–67.8)	67.0 (61.6–70.3)	66.7 (61.6–70.6)	1.09 (1.06–1.11), < 0.001
**Sex**				
Women	70,355 (98.40%)	54 (0.10%)	1098 (1.50%)	1.00 (1.00–1.00)
Men	76,559 (97.00%)	148 (0.20%)	2177 (2.80%)	2.48 (1.82–3.40), < 0.001
***H. pylori***				
Non‐tested/negative	102,426 (97.50%)	125 (0.10%)	2549 (2.40%)	1.00 (1.00–1.00)
Exposed	44,488 (98.20%)	77 (0.20%)	726 (1.60%)	1.43 (1.08–1.90), 0.004
**Country of birth**				
Israel	89,870 (97.90%)	89 (0.10%)	1818 (1.90%)	1.00 (1.00–1.00)
Immigrant Asia	9969 (97.20%)	22 (0.20%)	267 (2.60%)	2.20 (1.38–3.52), < 0.001
Immigrant Europe	9444 (97.60%)	20 (0.20%)	209 (2.20%)	2.13 (1.31–3.46), 0.002
Immigrant Former USSR	19,636 (97.30%)	48 (0.20%)	496 (2.50%)	2.46 (1.73–3.49), < 0.001
Immigrant Africa	17,995 (97.20%)	23 (0.10%)	485 (2.60%)	1.28 (0.80–2.02), 0.291
**Socioeconomic state**				
High	23,492 (98.50%)	31 (0.10%)	330 (1.40%)	1.00 (1.00–1.00)
Medium	94,178 (97.60%)	131 (0.10%)	2136 (2.20%)	1.04 (0.70–1.54), 0.824
Low	29,244 (97.10%)	40 (0.10%)	809 (2.70%)	1.03 (0.64–1.64), 0.922
**Smoking status**				
Never	83,318 (98.30%)	83 (0.10%)	1321 (1.60%)	1.00 (1.00–1.00)
Current/past	65,596 (96.80%)	119 (0.20%)	1954 (3.00%)	1.85 (1.40–2.45), < 0.001
**Anemia** ^a^				
No	124,173 (98.30%)	146 (0.10%)	1930 (1.50%)	1.00 (1.00–1.00)
Yes	22,741 (94.20%)	56 (0.20%)	1345 (5.60%)	2.01 (1.48–2.74), < 0.005
**Proton pump inhibitors** ^a^				
No	114,932 (98.10%)	141 (0.10%)	2095 (1.80%)	1.00 (1.00–1.00)
Yes	31,982 (96.30%)	61 (0.20%)	1180 (3.50%)	1.52 (1.13–2.07), 0.006
**Aspirin** ^a^				
No	106,103 (98.20%)	122 (0.10%)	1824 (1.70%)	1.00 (1.00–1.00)
Yes	40,811 (96.40%)	80 (0.20%)	1451 (3.40%)	1.68 (1.27–2.23), < 0.001
**NSAIDS** ^a^				
No	117,386 (98.00%)	166 (0.10%)	2613 (2.20%)	1.00 (1.00–1.00)
Yes	29,528 (97.40%)	36 (0.10%)	662 (1.90%)	0.86 (0.60–1.23), 0.413
**Anti‐platelet/coagulants** ^a^				
No	137,818 (98.0%)	187 (0.13%)	2620 (1.86%)	1.00 (1.00–1.00)
Yes	9096 (93.14%)	15 (0.15%)	655 (6.71%)	1.16 (0.68–1.96), 0.581

Abbreviations: FIT, fecal immunochemical test; IQR, interquartile range; NSAIDS, non‐steroidal anti‐inflammatory drugs; USSR, Union of Soviet Socialist Republics.

^a^ Within 6 months before the FIT date. *H. pylori* exposure was defined as either a positive stool antigen, a positive breath test, or receipt of a specific anti‐ *H. pylori* treatment.

### 3.3 Cost‐Effectiveness Analysis

We assessed the cost‐effectiveness of a one‐time EGD following a positive FIT compared with no additional screening. The base‐case analysis demonstrated an incremental cost‐effectiveness ratio (ICER) of USD 25,535 per QALY gained, which is well below the USD 50,000 threshold (Table S1). The incremental cost was USD 177 per person, reflecting both the procedure cost and downstream savings. Age‐specific analyses demonstrated increasing ICERs with age: approximately USD 14,000 per QALY at age 50 and USD 36,000 per QALY at age 70 (Table S2). Generalizability Analyses: To address concerns about transferability to different healthcare cost settings, we examined a wide range of EGD and treatment cost combinations. At EGD costs of USD 600 (71% higher than the base case), combined with treatment costs 50% lower than the base case, representing a scenario unfavorable to cost‐effectiveness, the ICER remained at USD 43,000 per QALY, still below the USD 50,000 threshold. When EGD costs increased to USD 800, the ICER rose to USD 55,000 per QALY, and at USD 1000 (representing very high‐cost settings), the ICER reached USD 78,000 per QALY. These analyses suggest that screening remains cost‐effective in most healthcare systems but may be marginal in settings with very high endoscopy costs (> USD 800) relative to treatment costs. (Table S3).

### 3.4 Scenario Analyses

Screening showed even more favorable cost‐effectiveness in high‐risk populations. Among *H. pylori‐*positive immigrants, who had an observed cancer incidence of 0.24% in our cohort, the ICER was USD 18,400 per QALY, substantially more cost‐effective than the base case (Table S4). When analyzing gastric cancer alone (excluding esophageal cancer, which comprised approximately 30% of cancers in our cohort), screening remained cost‐effective with an ICER of USD 35,800 per QALY. Age‐stratified analyses by 5‐year intervals confirmed cost‐effectiveness across all subgroups, with the most favorable ICERs in younger age groups (50–55 years: USD 14,000–16,500 per QALY) and acceptable ICERs even in the oldest eligible group (71–75 years: USD 38,000–40,000 per QALY). Of the 202 GEC cases diagnosed during 36 months of follow‐up, nearly half (48%) were detected between 19 and 36 months, supporting the assumption of 50% localized disease in the base‐case model. Comprehensive sensitivity analyses (Table S4, Figures S1–S5) confirmed the robustness of the findings. In particular, varying the proportion of cancers detected at a localized stage across a wide range consistently showed that screening remained cost‐effective. The tornado diagram (Figure S1) identified the probability of localized cancer, discount rate, and age as the most influential variables, with ICERs ranging from approximately USD 8500–77,000 per QALY.

### 3.5 Further Analysis

Among those with documented *H. pylori* exposure, the GEC incidence was 0.17% in both FIT‐positive (19/11,402) and FIT‐negative (58/33,889) individuals (*p* = 0.925). However, participants who received treatment for *H. pylori* had a higher incidence of GEC (0.18%; 73/40,874) than the non‐tested/tested‐negative group (0.12%), corresponding to an adjusted HR of 1.51 (95% CI, 1.14–2.02). In the cohort that was not tested or tested negative for *H. pylori* , there were 125 GEC cases. The incidence was 0.17% (45/26,307) in FIT‐positive individuals versus 0.10% (80/78,793) in FIT‐negative individuals, with an adjusted HR of 1.68 (95% CI, 1.17–2.43). Site‐specific stratification revealed that a positive FIT was significantly associated with gastric cancer (173 cases: 0.15% [57/37,709] in FIT‐positive vs. 0.10% [116/112,682] in FIT‐negative; adjusted HR 1.37, 95% CI, 1.00–1.89), but not esophageal cancer (29 cases: 0.018% [7/37,709] vs. 0.019% [22/112,682]; adjusted HR 0.86, 95% CI, 0.36–2.01). In a sensitivity analysis restricted to participants without anemia, 146 GEC cases were identified. The incidence was 0.15% (46/30,670) among FIT‐positive individuals and 0.10% (100/95,579) among FIT‐negative individuals, yielding an adjusted HR of 1.43 (95% CI, 1.01–2.03). For small bowel cancer, 11 cases were observed: 0.021% (8/37,709) in FIT‐positive vs. 0.003% (3/112,682) in FIT‐negative individuals, with a markedly increased risk (adjusted HR 7.98; 95% CI, 2.12–30.1).

## 4 Discussion

This matched cohort study evaluated the association between a positive FIT and the risk of GEC and assessed the cost‐effectiveness of adding a one‐time EGD to colonoscopy in FIT‐positive individuals.

### 4.1 Principal Findings

FIT positivity was associated with a modest but statistically significant increase in GEC risk (0.17% vs. 0.12%; adjusted HR 1.39, 95% CI 1.03–1.87). Despite the low absolute incidence, a one‐time EGD was cost‐effective across multiple scenarios (ICER USD 25,535/QALY) in our setting. Immigration—particularly from the former USSR—was independently associated with a higher risk (HR 2.46, 95% CI 1.73–3.49), and is even more cost‐effective (ICER of USD 18,400 per QALY). Current guidance reflects the need to balance benefits and resources: the ESGE suggests endoscopic screening for high‐risk populations and, in intermediate‐risk regions, where resources allow [1]. The AGA suggests screening for immigrants from high‐incidence regions and other high‐risk groups [2]. A recent systematic review/meta‐analysis supports the potential value of EGD screening in intermediate‐risk countries, especially when integrated with colorectal screening programs [3]. Our findings provide real‐world evidence consistent with such integrated strategies.

### 4.2 Comparison With Previous Studies

Our findings are consistent with earlier population‐based studies from the Netherlands and the United States, both of which reported an elevated risk of upper gastrointestinal cancer among FIT‐positive individuals but noted that the overall incidence remained below 1% [10, 11, 12]. A recent study from Portugal reported that approximately 51% of FIT‐positive individuals who were referred for colonoscopy also underwent upper endoscopy, and this was associated with increased early‐stage detection and improved survival [30]. Our study adds to this literature by using a matched cohort design (age, sex, *H. pylori* exposure) and evaluating additional determinants, including smoking, anemia, immigration background, medication use, and *H. pylori* treatment, providing a more comprehensive view of risk. While residual confounding and collinearity remain possible, the consistency across settings supports the robustness of the FIT‐GEC association.

### 4.3 Other Considerations

The association between PPI use and esophageal cancer may largely reflect confounding by indication, as PPIs are frequently prescribed to patients with gastroesophageal reflux disease (GERD) or Barrett's esophagus [31]. Regarding gastric cancer, the association between long‐term PPI use and increased risk, particularly among individuals treated for *H. pylori* infection, has been well documented [32]. PPIs may also reduce blood degradation in the upper gastrointestinal tract, thereby increasing the likelihood that occult bleeding will be detected by FIT [8]. Concerns have also been raised that anticoagulant use might increase the probability of FIT positivity in patients with upper gastrointestinal lesions [10]; however, no such association was observed in our cohort. Although the number of small bowel cancer cases was limited (*n* = 11), FIT positivity was significantly associated with this outcome. This is biologically plausible, as occult bleeding from small bowel tumors can be detected by FIT [11, 33].

### 4.4 Strengths and Limitations

Strengths of this study include the use of a large, real‐world matched cohort, which enhances internal validity by ensuring comparability of key baseline factors. Linkage to validated pharmacy and cancer registry data further strengthens the reliability of outcome ascertainment. Importantly, we integrated cost‐effectiveness modeling, which extends the clinical findings to policy‐relevant insights and provides a practical framework for decision‐making. In addition, the study incorporated multiple established risk determinants, such as smoking, treatment for *H. pylori* , country of birth, anemia, and PPI use, all of which are recognized contributors to GEC risk [34, 35, 36, 37]. The consistent alignment of these variables with our results both supports the robustness of our observations and validates the study's conclusions. Limitations include reliance on local incidence and cost structures (generalizability), lack of staging and premalignant conditions (e.g., gastric intestinal metaplasia, Barrett's esophagus), and categorical (nonquantitative) FIT results. Data on advanced colorectal adenomas were unavailable, which may have biased our results. However, given that the sensitivity of FIT for advanced adenomas is relatively low (≈20%–30%) [38], their exclusion is unlikely to fully explain the observed associations, though some bias cannot be excluded.

## 5 Conclusions

This age‐sex‐*H. pylori* exposure‐matched cohort study suggests that individuals with a positive FIT are at increased risk of GEC, with rates approaching those of *H. pylori* exposure and immigration from high‐risk countries. While the absolute incidence remains low, our findings, supported by cost‐effectiveness modeling, indicate that adding a one‐time EGD to colonoscopy in FIT‐positive individuals could be a reasonable and potentially cost‐effective strategy in healthcare systems where resources allow. Importantly, Israel is a diverse immigration country with subpopulations at a particularly elevated risk of gastric cancer, such as immigrants from the former USSR and parts of Asia, and exposure to *H. pylori* . This unique demographic context enhances the relevance of our findings and may support a tailored screening approach that balances local epidemiology, equity, and resource availability. Broader validation in other healthcare settings and prospective evaluation of personalized risk‐stratified strategies will be critical before routine implementation can be considered.

## Author Contributions

Conception and design: Z.L. Design: A.T., T.B. Data extraction: O.S.A., R.G.B., T.K. Analysis and interpretation of the data: Z.L., S.E., E.H.‐O., M.A.G., H.E.‐K. Drafting of the article: E.H.‐O., A.A.F., N.A.‐F., D.B. Critical revision of the article for important intellectual content: E.H.‐O., N.A.‐F., A.A.F. Cost effectiveness analysis: M.L. Final approval of the article: Z.L. All authors included in the authorship list approved the final version of this article.

## Funding

This work was supported by Clalit Health Research Fund and The Israeli Cancer Association Research Fund.

## Ethics Statement

The Ethics Committee of Clalit Health approved the study.

## Conflicts of Interest

The authors declare no conflicts of interest.

## Supporting information


**Table S1:** Base‐case cost‐effectiveness results.
**Table S2:** Age‐stratified cost‐effectiveness results.
**Table S3:** Generalizability analysis—cost‐effectiveness across different healthcare cost settings.
**Table S4:** Scenario analysis results.
**Figure S1:** Tornado diagram—One‐Way sensitivity analysis.
**Figure S2:** One‐Way sensitivity analysis—probability of localized cancer.
**Figure S3:** One‐Way sensitivity analysis—age at screening.
**Figure S4:** One‐Way sensitivity analysis—discount rate.
**Figure S5:** One‐Way sensitivity analysis—time horizon.

## Data Availability

The data that support the findings of this study are available on request from the corresponding author. The data are not publicly available due to privacy or ethical restrictions.
