# Supplementary material for: The Association of a Positive Fecal Immunochemical Test With the Risk of Gastroesophageal Cancer: An Age‐Sex‐H. Pylori Exposure Matched Cohort Study and Cost‐Effectiveness Analysis
Source: Helicobacter. 2026 Mar 23;31(2):e70120. doi: 10.1111/hel.70120 (PMC13009314; doi:10.1111/hel.70120)
Supplement: Supplementary file 1 — Table S1: Base‐case cost‐effectiveness results. Table S2: Age‐stratified cost‐effectiveness results. Table S3: Generalizability analysis—cost‐effectiveness across different healthcare cost settings. Table S4: Scenario analysis results. Figure S1: Tornado diagram—One‐Way sensitivity analysis. Figure S2: One‐Way sensitivity analysis—probability of localized cancer. Figure S3: One‐Way sensitivity analysis—age at screening. Figure S4: One‐Way sensitivity analysis—discount rate. Figure S5: One‐Way sensitivity analysis—time horizon. [file HEL-31-e70120-s001.docx]

**SUPPLEMENTARY MATERIAL**

**SUPPLEMENTARY TABLES**

**Supplementary Table S1: Base-Case Cost-Effectiveness Results**

| **Strategy** | **Cost (USD)** | **Incremental Cost (USD)** | **QALYs** | **Incremental QALYs** | **ICER (USD/QALY)** |
| --- | --- | --- | --- | --- | --- |
| No Screening | 4,157 | - | 12.3938 | - | - |
| One-time EGD | 4,334 | 177 | 12.4008 | 0.0070 | 25,535 |

**Supplementary Table S2: Age-Stratified Cost-Effectiveness Results**

| **Age at Screening** | **Cost (USD)** | **QALYs** | **ICER (USD/QALY)** |
| --- | --- | --- | --- |
| 50 | 4,521 | 15.82 | 14,000 |
| 55 | 4,447 | 14.28 | 16,500 |
| 60 | 4,392 | 13.21 | 19,800 |
| 65 (base case) | 4,334 | 12.40 | 25,535 |
| 70 | 4,268 | 11.18 | 36,200 |

**Supplementary Table S3: Generalizability Analysis - Cost-Effectiveness Across Different Healthcare Cost Settings**

| **EGD Cost (USD)** | **Treatment Cost Adjustment** | **ICER (USD/QALY)** | **Interpretation** |
| --- | --- | --- | --- |
| 200 | Base case | 18,000 | Very cost-effective |
| 200 | -50% | 22,000 | Very cost-effective |
| 200 | +50% | 14,000 | Very cost-effective |
| 300 | Base case | 22,000 | Cost-effective |
| 300 | -50% | 32,000 | Cost-effective |
| 300 | +50% | 16,000 | Cost-effective |
| 350 (base) | Base case | 25,535 | Cost-effective |
| 350 | -50% | 36,000 | Cost-effective |
| 350 | +50% | 18,000 | Cost-effective |
| 400 | Base case | 28,000 | Cost-effective |
| 400 | -50% | 39,000 | Cost-effective |
| 400 | +50% | 21,000 | Cost-effective |
| 600 | Base case | 38,000 | Cost-effective |
| 600 | -50% | 43,000 | Cost-effective |
| 600 | +50% | 28,000 | Cost-effective |
| 800 | Base case | 55,000 | Marginal |
| 800 | -50% | 62,000 | Marginal |
| 800 | +50% | 48,000 | Cost-effective |
| 1,000 | Base case | 78,000 | Not cost-effective |
| 1,000 | -50% | 88,000 | Not cost-effective |
| 1,000 | +50% | 68,000 | Marginal |

**Note:** Cost-effective defined as ICER <$50,000/QALY; Marginal: $50,000-75,000/QALY; Not cost-effective: >$75,000/QALY

**Supplementary Table S4: Scenario Analysis Results**

| **Scenario** | **Description** | **ICER (USD/QALY)** | **Change from Base Case** |
| --- | --- | --- | --- |
| **Base case** | All FIT-positive, age 65 | 25,535 | - |
| **High-risk populations** |  |  |  |
| H. pylori-positive immigrants | Cancer incidence 0.24% | 18,400 | -28% (more favorable) |
| **Age stratification (5-year intervals)** |  |  |  |
| Ages 50-55 | Youngest eligible cohort | 14,000-16,500 | -45% to -35% |
| Ages 56-60 | Young-middle age | 17,000-19,800 | -33% to -22% |
| Ages 61-65 | Middle age (includes base case) | 22,000-25,535 | -14% to base |
| Ages 66-70 | Older middle age | 28,000-33,000 | +10% to +29% |
| Ages 71-75 | Oldest eligible | 38,000-40,000 | +49% to +57% |
| **Cancer site** |  |  |  |
| Gastric cancer only | Excluding esophageal (~30% of cancers) | 35,800 | +40% |
| **Stage distribution at detection** |  |  |  |
| 30% localized (screening) | Lower screening effectiveness | 38,000 | +49% |
| 40% localized (screening) | Moderate screening effectiveness | 30,000 | +17% |
| 50% localized (screening) | Base case screening effectiveness | 25,535 | Base case |
| 60% localized (screening) | Higher screening effectiveness | 20,000 | -22% |
| 70% localized (screening) | Very high screening effectiveness | 15,000 | -41% |

**Interpretation:** All scenarios remained cost-effective (ICER <$50,000/QALY), with the most favorable cost-effectiveness in high-risk populations and younger age groups.

**Supplementary Figure S1: Tornado Diagram - One-Way Sensitivity Analysis**


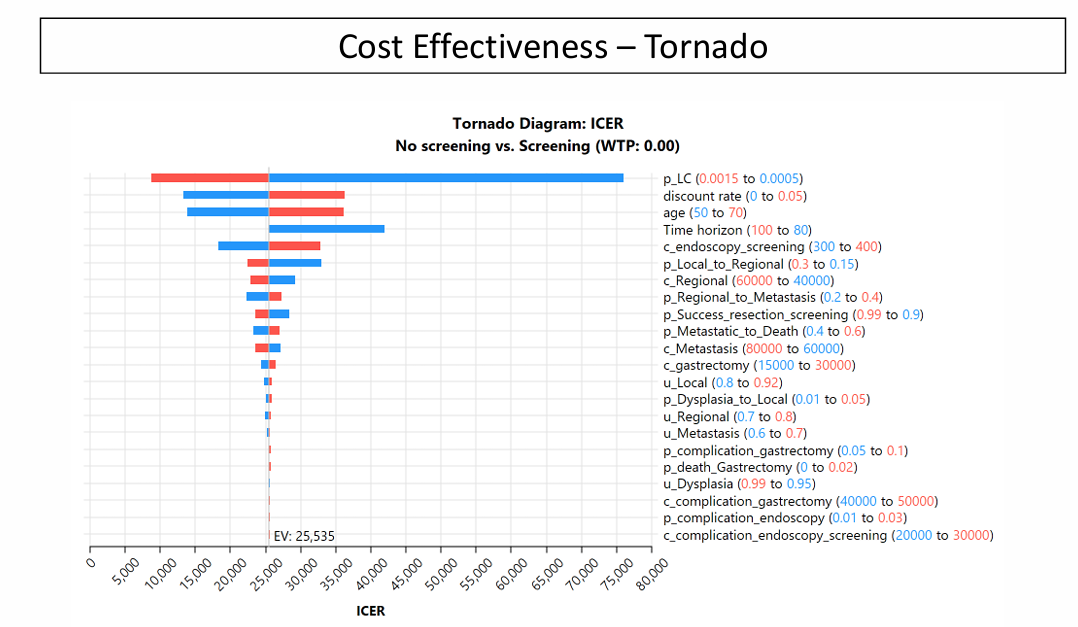


**Description:** Tornado diagram showing the impact of varying each model parameter across its plausible range on the incremental cost-effectiveness ratio (ICER). Parameters are ranked by magnitude of impact, with the most influential at the top. Bars extend from minimum (left) to maximum (right) ICER values when each parameter varies from its low to high value. The vertical line represents the base-case ICER of $25,535/QALY.

**Key Finding:** Probability of localized cancer (p_LC) is the most influential parameter, with ICERs ranging from approximately $8,500/QALY (high cancer incidence) to $77,000/QALY (low cancer incidence).

**Supplementary Figure S2: One-Way Sensitivity Analysis - Probability of Localized Cancer**


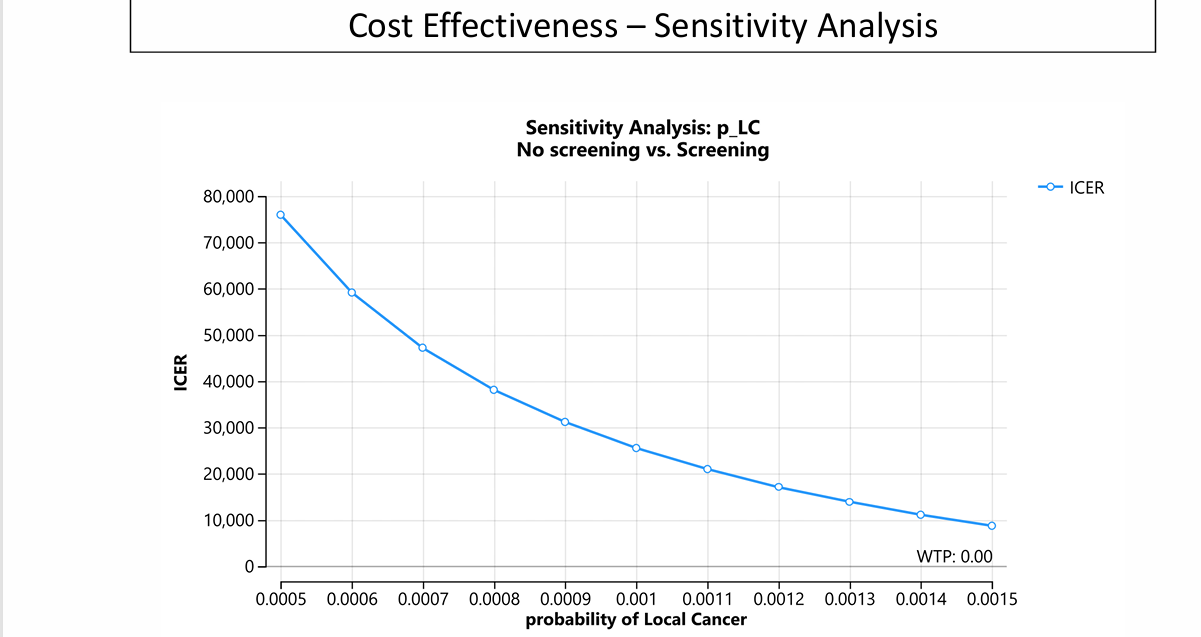


**Description:** Graph showing the relationship between probability of localized cancer (p_LC) on the x-axis (ranging from 0.0005 to 0.0015) and ICER (USD/QALY) on the y-axis. The curve demonstrates inverse relationship between cancer incidence and cost-effectiveness.

**Key Finding:** As cancer incidence increases from 0.0005 (50% of base case) to 0.0015 (150% of base case), the ICER decreases from approximately $77,000/QALY to $8,500/QALY, demonstrating that screening becomes increasingly cost-effective with higher cancer prevalence. At the observed cohort incidence (p_LC = 0.001), the ICER is $25,535/QALY.

**Supplementary Figure S3: One-Way Sensitivity Analysis - Age at Screening**


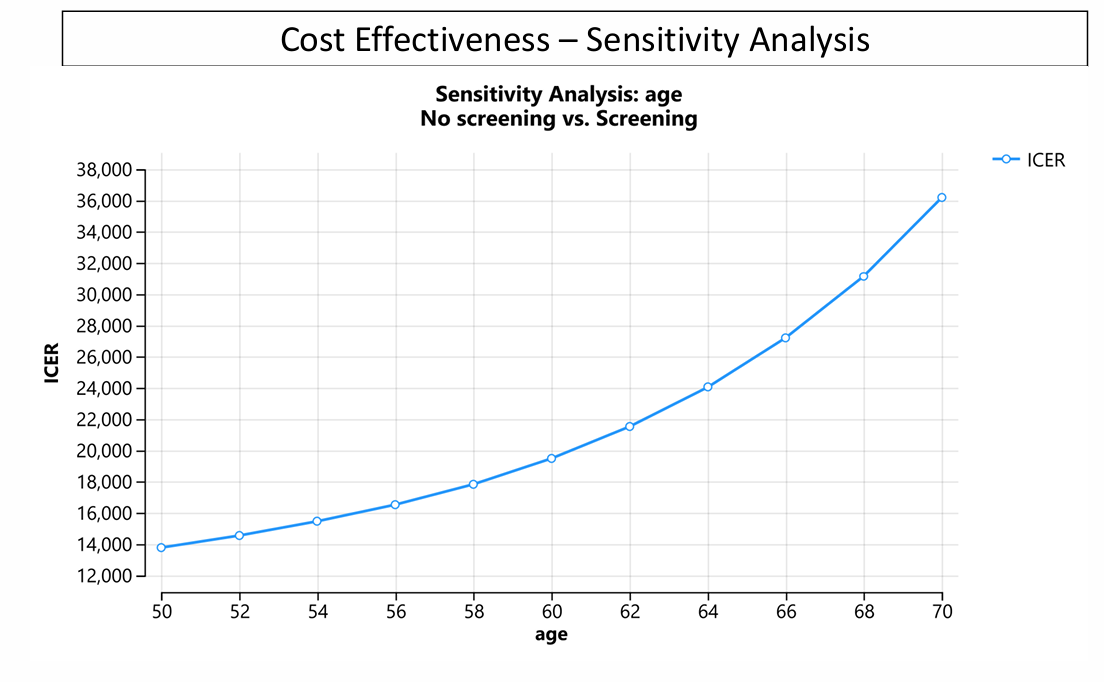


**Description:** Graph showing the relationship between age at screening (x-axis, 50-70 years) and ICER (USD/QALY, y-axis). The curve demonstrates increasing ICER with increasing age.

**Key Finding:** Cost-effectiveness decreases with age due to shorter life expectancy and competing mortality. ICER increases from $14,000/QALY at age 50 to $36,000/QALY at age 70. However, screening remains cost-effective across the entire eligible age range (all ICERs below $50,000/QALY threshold).

**Supplementary Figure S4: One-Way Sensitivity Analysis - Discount Rate**


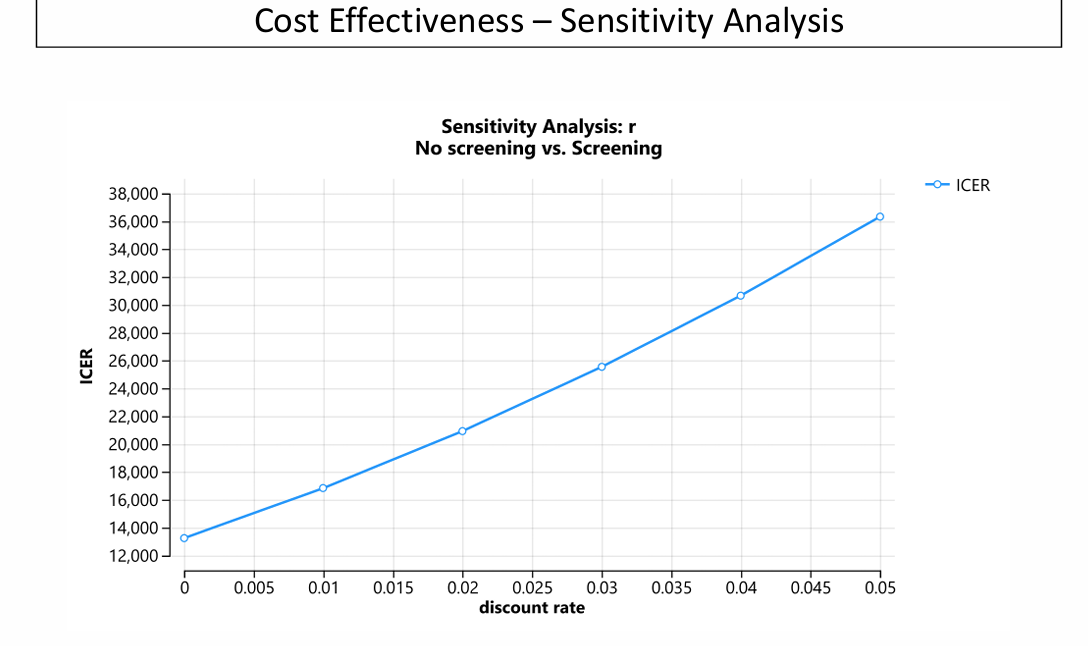


**Description:** Graph showing the relationship between annual discount rate (x-axis, 0% to 5%) and ICER (USD/QALY, y-axis). The curve demonstrates increasing ICER with increasing discount rate.

**Key Finding:** ICER increases from $13,000/QALY with no discounting (0%) to $36,000/QALY at 5% annual discounting. The base-case 3% discount rate yields an ICER of $25,535/QALY. Higher discount rates reduce the present value of future health benefits relative to immediate costs.

**Supplementary Figure S5: One-Way Sensitivity Analysis - Time Horizon**


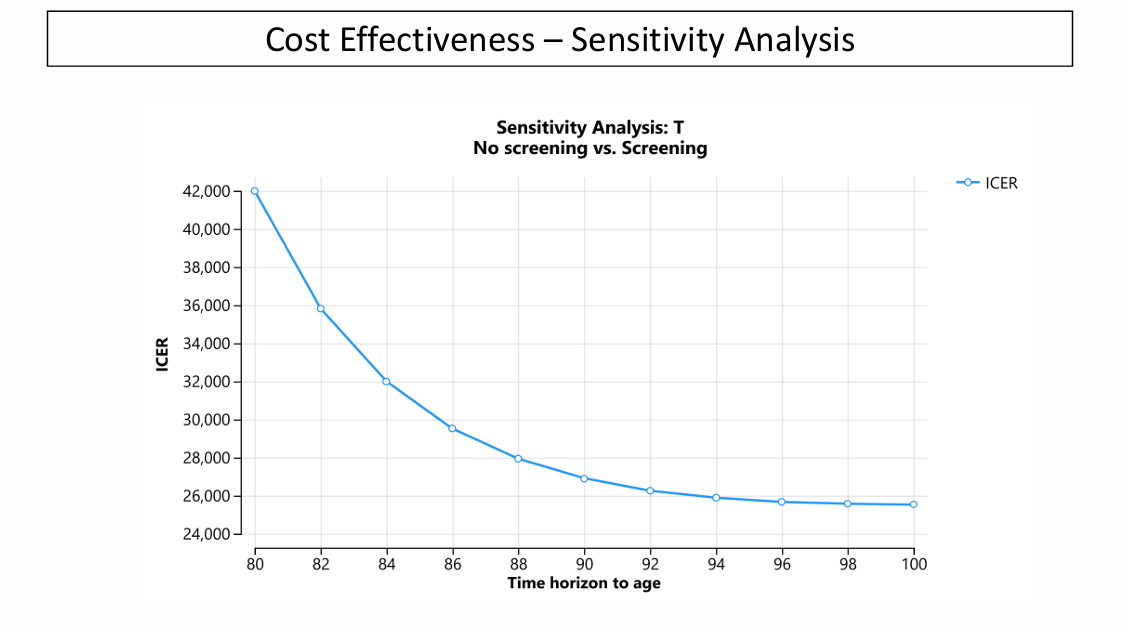


**Description:** Graph showing the relationship between time horizon (x-axis, 80-100 years) and ICER (USD/QALY, y-axis). The curve demonstrates decreasing ICER with longer time horizons.

**Key Finding:** ICER decreases from $42,000/QALY with an 80-year time horizon to $25,500/QALY with a 100-year horizon. Longer time horizons capture more lifetime benefits from cancer prevention and early detection. The model plateaus beyond 100 years as most individuals have died by this point.
